# Emerging Therapies for Non-Alcoholic Steatohepatitis (NASH): A Comprehensive Review of Pharmacological and Non-Pharmacological Approaches

**DOI:** 10.7759/cureus.69129

**Published:** 2024-09-10

**Authors:** Shradha P Kakde, Maham Mushtaq, Maryyam Liaqat, Husnain Ali, Muhammad Muaz Mushtaq, Muhammad Asad Sarwer, Sami Ullah, Muhammad Wali Hassan, Asma Khalid, Syed Faqeer Hussain Bokhari

**Affiliations:** 1 Internal Medicine, Mahatma Gandhi Mission Institute of Health Sciences, Aurangabad, IND; 2 Medicine and Surgery, King Edward Medical University, Lahore, PAK

**Keywords:** fxr agonists, lipotoxicity, liver fibrosis, nafld, nash, nonalcoholic fatty liver disease (nafld), non-alcoholic steatohepatitis, ppar agonists

## Abstract

Non-alcoholic steatohepatitis (NASH) has emerged as a significant global health concern, closely linked to the obesity epidemic and metabolic syndrome. This review explores emerging therapies for NASH that go beyond traditional lifestyle modifications. The complex pathophysiology of NASH, involving insulin resistance, lipotoxicity, oxidative stress, and chronic inflammation, offers multiple targets for therapeutic intervention. While lifestyle changes remain fundamental, their limitations in achieving sustained improvements highlight the need for effective pharmacological and interventional therapies. This review discusses novel pharmacological approaches, including farnesoid X receptor (FXR) agonists, peroxisome proliferator-activated receptor (PPAR) agonists, and agents addressing metabolic dysfunction, inflammation, and fibrosis. Promising candidates such as obeticholic acid, lanifibranor, and semaglutide are highlighted, along with combination therapies targeting multiple pathways simultaneously. Non-pharmacological interventions, including bariatric surgery, endoscopic bariatric and metabolic therapies, and innovative exercise regimens, are also examined for their potential in NASH management. Despite significant advancements, NASH drug development faces challenges due to the disease's complexity, patient heterogeneity, and stringent regulatory requirements. This review also addresses these limitations and explores future directions, including personalized medicine approaches, non-invasive diagnostic tools, and the potential of microbiome modulation and regenerative therapies. The evolving landscape of NASH research emphasizes the need for multidisciplinary approaches integrating advances in diagnostics, therapeutics, and digital health technologies. As the field progresses, the focus remains on developing more effective, personalized, and accessible strategies for preventing, diagnosing, and treating NASH, with the ultimate goal of improving outcomes for patients affected by this increasingly prevalent liver disease.

## Introduction and background

Non-alcoholic steatohepatitis (NASH) represents a significant health concern worldwide, emerging as a leading cause of chronic liver disease in developed countries [1]. NASH (now known as metabolic dysfunction-associated steatohepatitis [MASH]) is characterized by hepatic steatosis, inflammation, and hepatocellular injury, with the potential to progress to cirrhosis and hepatocellular carcinoma. As a more severe form of non-alcoholic fatty liver disease (NAFLD), NASH is closely associated with obesity, type 2 diabetes mellitus, and metabolic syndrome, reflecting the complex interplay between metabolic dysfunction and liver pathology [2]. The prevalence of NASH has been increasing in parallel with the global obesity epidemic. While precise global prevalence is challenging to determine due to the need for liver biopsy for a definitive diagnosis, estimates suggest that NASH affects 3-5% of the general population in Western countries [3]. This prevalence increases significantly with age and in high-risk groups, such as those with type 2 diabetes or severe obesity [3].

The natural history of NASH is variable, with some patients experiencing a relatively benign course while others progress rapidly to advanced fibrosis and its complications. Factors influencing disease progression include age, diabetes, and genetic predisposition [4]. The long-term consequences of NASH extend beyond liver-related morbidity and mortality, as it is also associated with an increased risk of cardiovascular disease, which remains the leading cause of death in these patients. Currently, lifestyle modifications focusing on weight loss through dietary changes and increased physical activity form the cornerstone of NASH management. A weight loss of 7-10% has been shown to improve liver histology, including resolution of steatohepatitis and regression of fibrosis in some patients [5]. However, achieving and maintaining this degree of weight loss is challenging for many individuals, with success rates in clinical practice often falling short of those observed in controlled trials. Moreover, even when successful, lifestyle interventions alone may be insufficient for patients with advanced disease or those with rapid progression. This limitation underscores the critical need for effective pharmacological and interventional therapies to address the growing burden of NASH.

The pathogenesis of NASH involves multiple parallel hits, including insulin resistance, hyperuricemia, hypertriglyceridemia, lipotoxicity, oxidative stress, and chronic inflammation, culminating in hepatocyte injury and fibrosis [6]. This complex pathophysiology offers numerous potential targets for therapeutic intervention. Over the past decade, significant efforts have been directed toward developing pharmacological agents that can effectively halt or reverse the progression of NASH. Despite these efforts, as of early 2024, there are no FDA-approved medications specifically for the treatment of NASH [7]. Several promising agents have failed in late-stage clinical trials (for example, elafibranor, cenicriviroc, and selonsertib), highlighting the challenges in drug development for this complex disease [8]. However, the pipeline of potential therapies remains robust, with numerous compounds in various stages of clinical development targeting different aspects of NASH pathogenesis.

This review aims to explore the emerging therapies for NASH that go beyond lifestyle modifications. We will discuss novel pharmacological approaches targeting key pathways involved in NASH progression, including farnesoid X receptor (FXR) agonists, peroxisome proliferator-activated receptor (PPAR) agonists, and agents addressing metabolic dysfunction, inflammation, and fibrosis. Additionally, we will examine non-pharmacological interventional strategies that show promise in NASH management. Understanding these emerging therapies is crucial for clinicians managing patients with NASH, as it provides insight into potential future treatment options and helps in guiding current management decisions. Furthermore, this knowledge is essential for researchers and pharmaceutical developers working to address the unmet needs of NASH therapy. As we delve into these emerging therapies, it is important to recognize that the field of NASH treatment is rapidly evolving. While some approaches show significant promise, others may face challenges in demonstrating long-term efficacy and safety. The ultimate goal remains to develop safe, effective, and accessible treatments that can significantly improve outcomes for patients with NASH, reducing the burden of advanced liver disease and associated complications.

## Review

Pathophysiology and therapeutic targets

The pathophysiology of NASH is complex and multifactorial, involving several interconnected mechanisms that contribute to liver injury, inflammation, and fibrosis. Understanding these pathways is crucial for identifying potential therapeutic targets.

Insulin resistance plays a central role in the development and progression of NASH. In the insulin-resistant state, there is increased lipolysis in adipose tissue, leading to an influx of free fatty acids (FFAs) to the liver [9]. Simultaneously, hyperinsulinemia promotes de novo lipogenesis in hepatocytes [10]. The combination of these processes results in hepatic lipid accumulation, a hallmark of NASH. Excess lipids, particularly saturated fatty acids, can induce lipotoxicity in hepatocytes. This lipotoxicity triggers various cellular stress responses, including endoplasmic reticulum (ER) stress and activation of inflammatory pathways. The accumulation of toxic lipid species, such as ceramides and diacylglycerols, further exacerbates insulin resistance, creating a vicious cycle. Targeting insulin resistance and lipotoxicity has been a focus of several therapeutic approaches. Insulin sensitizers, such as thiazolidinediones, aim to improve insulin sensitivity by reducing the influx of FFAs to the liver and decreasing hepatic lipogenesis. Metabolic regulators, including farnesoid X receptor (FXR) agonists and fibroblast growth factor 19 (FGF19) analogs, modulate hepatic metabolism, thereby reducing lipogenesis and enhancing insulin sensitivity [11]. Additionally, lipid-lowering agents like acetyl-CoA carboxylase (ACC) inhibitors directly reduce hepatic lipid content, addressing the lipotoxicity component of NASH [12].

Oxidative stress is a key feature of NASH pathogenesis. The accumulation of lipids in hepatocytes leads to increased β-oxidation, which can overwhelm the mitochondrial electron transport chain, resulting in the production of reactive oxygen species (ROS) [13]. This oxidative stress can damage cellular components, including mitochondrial DNA, proteins, and lipids, leading to mitochondrial dysfunction. Impaired mitochondrial function further exacerbates oxidative stress and compromises ATP production, contributing to hepatocyte injury and death. The release of mitochondrial damage-associated molecular patterns (DAMPs) can trigger inflammatory responses, linking oxidative stress to hepatic inflammation [14]. Therapeutic approaches targeting oxidative stress and mitochondrial dysfunction include several promising strategies. Antioxidants, particularly those designed to accumulate specifically in mitochondria, are being explored due to the limited efficacy of general antioxidants. Mitochondrial function enhancers, such as peroxisome proliferator-activated receptor (PPAR) agonists, aim to improve mitochondrial function and biogenesis, potentially mitigating oxidative stress [15]. Additionally, apoptosis signal-regulating kinase 1 (ASK1) inhibitors are being investigated to reduce oxidative stress-induced cell death and inflammation [16].

Chronic inflammation is a defining feature of NASH that distinguishes it from simple steatosis [3]. Lipotoxicity, oxidative stress, and the release of DAMPs activate inflammatory pathways in hepatocytes and immune cells. This leads to the production of pro-inflammatory cytokines and chemokines, which recruit and activate various immune cells in the liver. Persistent inflammation, along with ongoing hepatocyte injury, activates hepatic stellate cells (HSCs), the primary fibrogenic cells in the liver. Activated HSCs transform into myofibroblasts, producing excessive extracellular matrix proteins and leading to liver fibrosis [17-19]. Therapeutic targets in this domain include several innovative strategies. Anti-inflammatory agents, such as inhibitors of C-C chemokine receptors, are designed to reduce hepatic inflammation by blocking specific inflammatory pathways. Antifibrotic agents focus on directly targeting HSC activation or promoting the resolution of fibrosis, with examples including galectin-3 inhibitors and lysyl oxidase-like 2 (LOXL2) inhibitors [20,21]. Additionally, combination approaches are being explored to address the interconnected nature of these pathways, aiming to target multiple aspects of NASH pathogenesis simultaneously.

Pharmacological therapies

The landscape of pharmacological therapies for non-alcoholic steatohepatitis (NASH) has expanded significantly in recent years, with several novel agents targeting various aspects of the disease pathogenesis.

Farnesoid X receptor (FXR) agonists have emerged as a leading class of drugs in NASH treatment [22]. FXR is a nuclear receptor highly expressed in the liver and intestine, playing a crucial role in bile acid homeostasis and lipid and glucose metabolism. Obeticholic acid, a semisynthetic bile acid analog and potent FXR agonist has shown promising results in NASH trials [23]. In the REGENERATE study, obeticholic acid demonstrated significant improvement in liver fibrosis without worsening NASH [24]. However, concerns about pruritus and lipid profile alterations have been noted [25]. Other FXR agonists, such as cilofexor and tropifexor, are also in clinical development, aiming to maintain efficacy while minimizing side effects.

Peroxisome proliferator-activated receptor (PPAR) agonists represent another important class of drugs in NASH therapy. PPARs are nuclear receptors involved in regulating metabolism, inflammation, and fibrosis [26]. Pioglitazone, a PPAR-γ agonist initially developed for diabetes, has shown efficacy in improving NASH histology. However, its use is limited by side effects such as weight gain and bone loss [27]. Newer agents targeting multiple PPAR subtypes are under investigation [28]. Lanifibranor, a pan-PPAR agonist, has shown promise in phase 2 trials, demonstrating improvement in NASH resolution and fibrosis regression [29]. The potential advantage of pan-PPAR agonists lies in their ability to address multiple aspects of NASH pathogenesis simultaneously. Dual and pan-PPAR agonists, such as elafibranor, are being developed to harness the beneficial effects of various PPAR isoforms [30]. These agents aim to improve insulin sensitivity, reduce lipotoxicity, and attenuate inflammation in NASH.

Glucagon-like peptide-1 (GLP-1) receptor agonists, initially developed for type 2 diabetes and obesity, have garnered interest in NASH treatment. Semaglutide, a long-acting GLP-1 analog, has shown promising results in early-phase NASH trials [31]. GLP-1 receptor agonists promote weight loss, improve insulin sensitivity, and may have direct effects on hepatic lipid metabolism. Their ability to address multiple components of the metabolic syndrome makes them attractive candidates for NASH therapy.

Thyroid hormone receptor-β (THR-β) agonists represent a novel approach to NASH treatment. Resmetirom, a selective THR-β agonist, has demonstrated the ability to reduce hepatic fat content and improve lipid profiles in clinical trials [32,33]. By targeting the liver-specific THR-β, these agents aim to harness the beneficial effects of thyroid hormone on lipid metabolism while minimizing systemic side effects associated with thyroid hormone excess.

Acetyl-CoA carboxylase (ACC) inhibitors target de novo lipogenesis in the liver. By inhibiting ACC, these drugs aim to reduce hepatic fat accumulation and improve insulin sensitivity. Firsocostat, an ACC inhibitor, has shown efficacy in reducing liver fat in early-phase trials [34,35]. However, its development as a monotherapy has been challenged by concerns about triglyceride elevations. Current investigations are focusing on its potential in combination therapies.

Apoptosis signal-regulating kinase 1 (ASK1) inhibitors, such as selonsertib, have been explored as potential antifibrotic agents in NASH [36]. ASK1 is a key mediator of oxidative stress-induced hepatocyte death and fibrosis. While initial results were promising, phase 3 trials of selonsertib did not meet their primary endpoints in patients with advanced fibrosis due to NASH [36,37]. This highlights the challenges in developing effective antifibrotic therapies for advanced stages of the disease.

The complexity of NASH pathogenesis has led to increased interest in combination therapies. The rationale is that targeting multiple pathways simultaneously may yield superior efficacy compared to monotherapies. Several combinations are being explored, including FXR agonists with ACC inhibitors and PPAR agonists with FXR agonists [38,39]. Early results from these combination approaches have been encouraging, showing additive or synergistic effects on NASH parameters [39]. While these emerging therapies show promise, it is important to note that NASH drug development has faced significant challenges. Several agents that showed initial promise have failed in late-stage trials, highlighting the complexity of the disease and the difficulties in translating preclinical and early clinical success to definitive phase 3 outcomes. Safety considerations, particularly for long-term use, remain a critical aspect of NASH therapy development.

As the field progresses, the focus is not only on developing effective therapies but also on identifying the right patients for each treatment approach. The heterogeneity of NASH underscores the need for personalized treatment strategies. Biomarkers and non-invasive diagnostic tools are being developed in parallel to help stratify patients and monitor treatment response, potentially allowing for more targeted and effective use of these emerging therapies.

Non-pharmacological therapies

While pharmacological approaches to NASH treatment continue to evolve, non-pharmacological interventional therapies have gained significant attention as potential alternatives or adjuncts to medical management. These interventions primarily target weight loss and metabolic improvement, which are fundamental to NASH resolution and fibrosis regression.

Bariatric surgery has emerged as a potent intervention for NASH, particularly in patients with obesity [40]. The dramatic weight loss achieved through bariatric procedures can lead to significant improvements in liver histology. Roux-en-Y gastric bypass (RYGB) and sleeve gastrectomy are the most commonly studied procedures in the context of NASH [41]. Multiple studies have demonstrated that bariatric surgery can result in NASH resolution and fibrosis regression in a substantial proportion of patients. The metabolic benefits of bariatric surgery, including improvements in insulin sensitivity and lipid profiles, contribute to its efficacy in NASH. However, it is crucial to note that bariatric surgery carries its own risks and is not suitable for all patients. Patient selection, timing of intervention, and long-term follow-up are critical considerations in using bariatric surgery for NASH management [42-44].

Endoscopic bariatric and metabolic therapies (EBMTs) represent a less invasive alternative to traditional bariatric surgery. These procedures aim to achieve weight loss and metabolic improvements through endoscopic techniques. Intragastric balloons and endoscopic sleeve gastroplasty are among the most studied EBMTs in the context of NASH [43,45]. Intragastric balloons, which involve the placement of a saline-filled balloon in the stomach to induce satiety, have shown promise in improving liver enzymes and reducing liver fat content in short-term studies. Endoscopic sleeve gastroplasty, a procedure that reduces the size of the stomach using endoscopic suturing, has also demonstrated efficacy in weight loss and improvement in NASH parameters [46]. While these techniques are generally associated with fewer complications compared to bariatric surgery, their long-term efficacy and durability in NASH management are still under investigation.

Lifestyle interventions remain the cornerstone of NASH management, but novel approaches are being explored to enhance their efficacy. Structured lifestyle intervention programs, often involving a multidisciplinary team of dietitians, exercise physiologists, and behavioral therapists, have shown superior results compared to standard dietary advice. These programs typically aim for a weight loss of 7-10%, which has been associated with significant improvements in NASH histology. [47] The challenge lies in achieving and maintaining this degree of weight loss in real-world settings. To address this, digital health interventions and telemedicine approaches are being investigated to provide more accessible and sustained support for lifestyle modifications in NASH patients.

Exercise-based interventions, independent of weight loss, have shown promise in improving NASH parameters. Both aerobic and resistance training have demonstrated benefits in reducing liver fat content and improving insulin sensitivity [48,49]. High-intensity interval training (HIIT) has gained particular interest due to its time efficiency and potent metabolic effects. Studies have shown that HIIT can significantly reduce liver fat and improve liver enzyme profiles in NASH patients, even in the absence of significant weight loss [50,51]. The optimal type, intensity, and duration of exercise for NASH management are still subjects of ongoing research.

Emerging non-pharmacological approaches include methods to modulate the gut microbiome, such as fecal microbiota transplantation (FMT). While still in the early stages of investigation for NASH, FMT has shown potential in altering the gut microbiome composition and improving metabolic parameters in small studies [52-54]. However, larger, well-designed trials are needed to establish its efficacy and safety in NASH management.

As research in non-pharmacological interventions for NASH progresses, the focus is increasingly on personalized approaches. Tailoring interventions based on individual patient characteristics, such as the degree of obesity, presence of comorbidities, and stage of liver disease, is likely to yield the best outcomes. Furthermore, combining these interventional approaches with pharmacological therapies may offer synergistic benefits, potentially leading to more effective NASH management strategies.

Limitations and challenges

The development of effective pharmacological therapies for NASH has proven to be a complex and challenging endeavor. Despite significant advancements in understanding the pathophysiology of NASH and the identification of numerous potential therapeutic targets, the field has faced several setbacks in clinical trials.

One of the primary challenges in NASH drug development is the complexity of the disease itself. NASH is a multifactorial condition involving intricate interactions between metabolic dysfunction, inflammation, and fibrosis. This complexity means that targeting a single pathway may be insufficient to achieve clinically meaningful improvements. Many drugs that have shown promise in preclinical studies or early-phase trials have failed to demonstrate efficacy in larger, late-stage clinical trials [7,55]. This highlights the difficulty in translating mechanistic insights into effective therapies and underscores the need for a more comprehensive approach to NASH treatment. The heterogeneity of NASH patients presents another significant challenge. NASH can occur in individuals with varying degrees of obesity, insulin resistance, and liver fibrosis. This heterogeneity makes it difficult to design clinical trials that can adequately capture the efficacy of a drug across the entire spectrum of NASH patients. Moreover, the response to treatment can vary significantly among patients, further complicating the assessment of drug efficacy. Efforts are ongoing to identify biomarkers and develop patient stratification strategies that can help predict treatment responses and tailor therapies to specific patient subgroups.

The regulatory landscape for NASH drug approval has also posed challenges. The U.S. Food and Drug Administration (FDA) and other regulatory agencies have set stringent criteria for demonstrating efficacy in NASH trials [56]. These typically include histological endpoints such as NASH resolution without worsening of fibrosis or improvement in fibrosis without worsening of NASH. Achieving these endpoints has proven difficult for many drug candidates. Furthermore, the requirement for paired liver biopsies to assess these histological endpoints introduces additional complications, including patient discomfort, sampling variability, and the risk of complications associated with the procedure. The long-term nature of NASH progression presents challenges in clinical trial design and execution. Demonstrating meaningful improvements in hard clinical outcomes, such as prevention of cirrhosis or reduction in liver-related mortality, would require trials of extended duration, often spanning several years. Such long-term trials are expensive, logistically challenging, and may suffer from high dropout rates. As a result, many trials rely on surrogate endpoints, which may not always translate to long-term clinical benefits.

Despite these challenges, the field of NASH drug development continues to evolve. There is a growing recognition of the need for combination therapies that target multiple aspects of NASH pathogenesis. Novel trial designs, including adaptive designs and the use of composite endpoints, are being explored to enhance the efficiency of clinical development. Additionally, efforts are underway to develop and validate non-invasive biomarkers and imaging techniques that could serve as surrogate endpoints in clinical trials. The challenges in NASH drug development underscore the complexity of the disease and the need for innovative approaches. As our understanding of NASH pathogenesis continues to grow and as new technologies and methodologies emerge, there is cautious optimism that these challenges can be overcome, leading to the development of effective therapies for this increasingly prevalent liver disease.

Future directions

The field of NASH research and treatment is rapidly evolving, with several promising avenues emerging for future development. As our understanding of the disease pathogenesis deepens and technology advances, new approaches to diagnosis, treatment, and management of NASH are on the horizon. Personalized medicine approaches are likely to play an increasingly important role in NASH management. The heterogeneity of NASH patients in terms of disease progression, risk factors, and treatment response underscores the need for tailored therapeutic strategies. Advances in genomics, proteomics, and metabolomics are enabling the identification of biomarkers that may predict disease progression and treatment response. These biomarkers, combined with clinical parameters, could allow for more precise patient stratification and treatment selection. For instance, genetic variants associated with NASH progression, such as PNPLA3 and TM6SF2, may guide risk assessment and treatment intensity [57]. Future research will likely focus on developing comprehensive risk prediction models that integrate genetic, metabolic, and environmental factors to guide personalized treatment plans.

The development of non-invasive diagnostic tools and biomarkers remains a critical area for future research. While current non-invasive methods such as elastography and serum biomarker panels have improved NASH diagnosis and staging, there is still a need for more accurate and widely available tools [58]. Advanced imaging techniques, including multiparametric MRI and novel PET tracers, are being investigated for their ability to assess liver fat, inflammation, and fibrosis non-invasively [59]. Liquid biopsy approaches, analyzing circulating biomarkers such as cell-free DNA, extracellular vesicles, and circulating RNA, hold promise for non-invasive diagnosis and monitoring of NASH [60]. The successful development of these tools could revolutionize NASH clinical trials and patient care, allowing for earlier diagnosis and more frequent monitoring of disease progression and treatment response.

Combination therapy approaches are likely to become more prevalent in NASH treatment. Given the multifactorial nature of NASH, targeting multiple pathways simultaneously may yield superior efficacy compared to monotherapies. Future clinical trials will likely focus on rational combinations of drugs with complementary mechanisms of action. For example, combining agents that improve metabolic parameters with those that have direct anti-inflammatory or antifibrotic effects may provide synergistic benefits. The challenge will be in optimizing these combinations to maximize efficacy while maintaining an acceptable safety profile.

The role of the gut microbiome in NASH pathogenesis and treatment is an area of intense research that is likely to yield new therapeutic approaches. As our understanding of the gut-liver axis deepens, interventions targeting the microbiome, such as prebiotics, probiotics, and microbiome modulators, may emerge as novel treatment strategies. Fecal microbiota transplantation, while still in the early stages of investigation for NASH, represents another potential avenue for microbiome-based therapies.

Digital health technologies and artificial intelligence (AI) are poised to transform NASH management [61]. Wearable devices and smartphone apps could enable more comprehensive and continuous monitoring of lifestyle factors relevant to NASH, such as diet, physical activity, and sleep patterns. AI algorithms could integrate these data with clinical and laboratory parameters to provide personalized recommendations and early warning of disease progression. In clinical trials, these technologies could enhance patient monitoring and provide richer data sets for analysis.

Cell-based therapies and regenerative medicine approaches represent an exciting frontier in NASH treatment, particularly for advanced stages of the disease. Stem cell therapies, including mesenchymal stem cells and hepatic progenitor cells, are being investigated for their potential to promote liver regeneration and reduce fibrosis [62,63]. While still in the early stages, these approaches could offer new hope for patients with advanced NASH and limited treatment options.

As research progresses, a greater emphasis on prevention strategies is likely to emerge. Identifying individuals at high risk for NASH and implementing early interventions could significantly reduce the burden of the disease. This may involve a combination of genetic screening, metabolic profiling, and lifestyle interventions tailored to individual risk profiles. The future of NASH management will likely involve a multidisciplinary approach, integrating advances in diagnostics, therapeutics, and digital health technologies. As these various strands of research progress, the goal remains to develop more effective, personalized, and accessible strategies for preventing, diagnosing, and treating NASH, ultimately improving outcomes for patients affected by this increasingly prevalent liver disease.

## Conclusions

The landscape of NASH management is evolving, driven by a better understanding of its complex pathophysiology and the urgent need for effective therapies. Emerging pharmacological agents targeting key pathways, such as FXR agonists, PPAR agonists, and metabolic regulators, offer hope for improved outcomes. Non-pharmacological interventions, including bariatric procedures and innovative lifestyle modifications, show potential in addressing underlying metabolic dysfunctions. Future NASH management will likely involve personalized, multifaceted approaches integrating pharmacological, interventional, and lifestyle therapies. Accurate, non-invasive diagnostic tools and biomarkers are crucial for early diagnosis and treatment monitoring. Combination therapies targeting multiple aspects of NASH pathogenesis are expected to achieve significant improvements. Emphasis on prevention and early intervention in high-risk individuals, alongside emerging technologies like digital health solutions and AI, will enhance patient care. Novel approaches such as microbiome modulation and regenerative therapies offer exciting prospects. Ongoing research and collaboration are vital for translating advancements into improved clinical outcomes.
